# Area of hydronephrosis is a useful predictive factor of impacted ureteral stones

**DOI:** 10.1007/s00240-023-01526-3

**Published:** 2024-04-02

**Authors:** Yuya Iwahashi, Yasuo Kohjimoto, Ryusuke Deguchi, Takahito Wakamiya, Shimpei Yamashita, Isao Hara

**Affiliations:** Department of Urology, Wakayama Medical University, 811-1, Kimiidera, Wakayama City, Wakayama 641-0012 Japan

**Keywords:** Area of hydronephrosis, Ureteral wall thickness, Impacted stone, Ureteroscopic lithotripsy

## Abstract

Ureteroscopic lithotripsy for impacted stones is challenging, but it is important to predict impacted stones preoperatively. Hydronephrosis, which is evaluated by a grading system, is often apparent in impacted stones. However, the currently used grading system is a qualitative evaluation. We, therefore, focused on a *quantitative* evaluation: the area of hydronephrosis. The aim of this study was to investigate whether the area of hydronephrosis could predict impacted stones more accurately than Society for Fetal Urology grade. We retrospectively identified 160 patients who underwent ureteroscopic lithotripsy for ureteral stones at our hospital between January 2014 and April 2022. Impacted stones were defined as stones fixed to the ureteral wall that could not be moved by means of ureteroscopic manipulation or water pressure. Of the 160 patients, 54 (33.8%) had impacted stones. Comparing patient characteristics, there were significant differences in stone size, ureteral wall thickness, Society for Fetal Urology grade, renal pelvic width and area of hydronephrosis (all *P* < 0.01). Receiver operating characteristic analysis showed that area of hydronephrosis was the more significant predictive value (area under the curve 0.781) compared with Society for Fetal Urology grade (area under the curve 0.676, *P* < 0.01). Multivariate analysis revealed that significant independent predictive factors of impacted stones were thicker ureteral wall thickness and larger area of hydronephrosis (both *P* < 0.01). The area of hydronephrosis and ureteral wall thickness were significant predictors of impacted stones in patients undergoing ureteroscopic lithotripsy for ureteral stones. These factors may be useful for selecting the treatment and preoperative settings.

## Introduction

An impacted stone is one which remains in the same position for a prolonged time and causes ureteral obstruction, although there is not currently definition in the literature [1, 2]. Confirmation of impacted stones can be done during ureteroscopy, showing adherence to the ureteral wall. When ureteroscopic lithotripsy (URSL) is performed on impacted stones, ureteral edema and polyps are often detected and these can lead to subsequent bleeding, possible ureteral injury, and future ureteral stricture [3, 4]. Patients with impacted stones are, therefore, considered to be at higher risk of ureteroscopic complications and are less likely to be stone free [5–7]. Moreover, impacted stones have been shown to negatively affect the success rate of laser endoureterotomy of ureteral strictures [8]. As an alternative approach to impacted stones, antegrade URSL has been suggested instead of retrograde URSL [9, 10]. Preoperative prediction of impacted stones seems to be very important.

Many researchers have attempted to reveal the predictive factors of impacted stones. Ureteral wall thickness (UWT) was reported by Sarica et al. to be a useful predictor that is measured at the site of the impacted stone using non-contrast computed tomography (NCCT) [11]. As well as UWT, age and stone location were suggested by Yoshida et al. to be strongly associated with impacted stones [7]. We recently reported that the ratio of CT attenuation of the ureter above/below ureteral stones is closely related to the presence of an impacted stone [2]. Hydronephrosis has also been reported to have a part in prediction [2, 12, 13]; it is related to the degree of obstruction by ureteral stones. It seems that the more severe the obstruction of the ureter becomes, the worse the degree of hydronephrosis will be. Hydronephrosis, which is evaluated by grading system (e.g., SFU grade), has often been apparent in previous reports [2, 12, 14]. However, the grading system is based upon qualitative factors. In the current study, we therefore, focused on the area of hydronephrosis (AH), which is a quantitative factor. To the best of our knowledge, no previous reports have quantitatively assessed hydronephrosis. The aim of this study is to investigate whether the AH in modified coronal images of NCCT can accurately predict impacted stones.

## Materials and methods

### Patients

We retrospectively reviewed the 213 patients who underwent URSL for ureteral stones between January 2014 and April 2022 at Wakayama Medical University, Wakayama, Japan. Patients who had kidney stones only (and thus were without ureteral stones) were not included. Exclusion criteria were renal anomalies including ureteropelvic junction obstruction, any previous intervention, multiple ureteral stones and inadequate data or NCCT images. Fifty-three patients met exclusion criteria. Finally, 160 patients were included in this study, all of whom underwent pretreatment NCCT. These patients all provided written informed consent to inclusion in the study, and ethical approval was obtained from the Wakayama Medical University Institutional Review Board (No.3487).

### Data collection

**Fig. 1:**
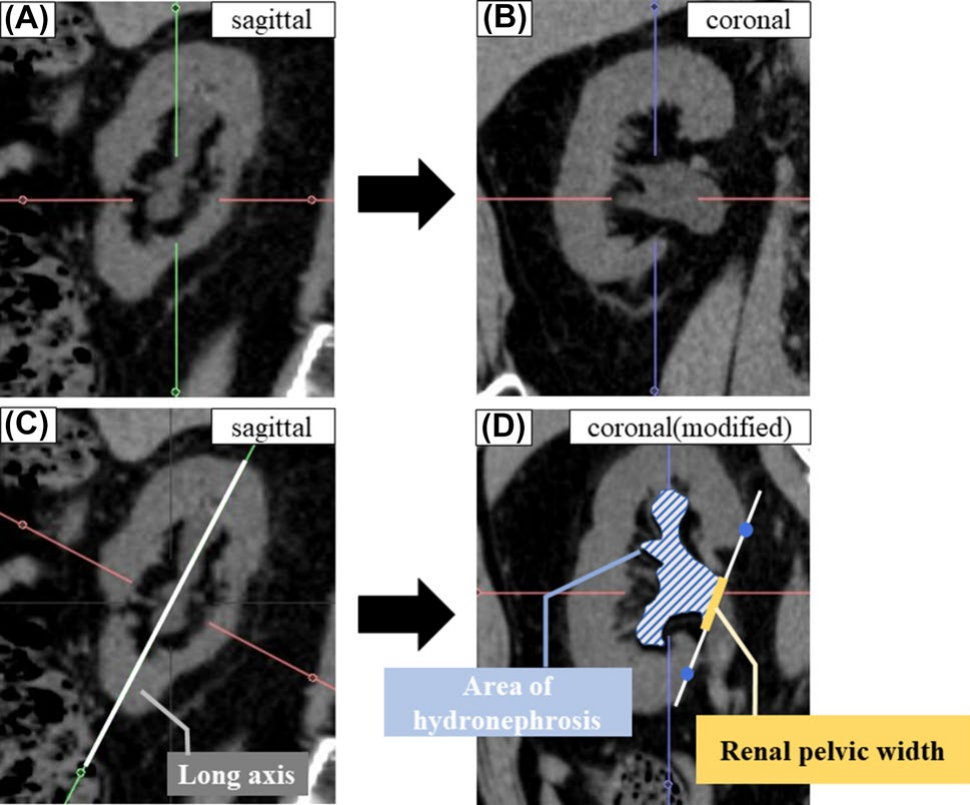
Measurements methods of the area of hydronephrosis (AH) and the renal pelvic width (RPW) from NCCT image. The long axis of the kidney was set by sagittal image (**C**). A modified coronal image was created by correction of the long axis (**D**). **A** and **B** show normal NCCT images of the same slice (not modified by the long axis). The AH was measured centrally from the line, bordered by the renal parenchyma edges in modified coronal image. The RPW was measured by the distance of the line crossing the ureter (**D**)

Patient characteristics were retrospectively collected from medical records. Operative factors were assessed by videos. Patient characteristics included sex, age, body mass index, Eastern Cooperative Oncology Group performance status. Clinical data included stone size, location, three-dimensional mean stone density (3D-MSD), UWT, Society for Fetal Urology grade (SFU grade), the renal pelvic width (RPW) and the AH. Stone size was measured at maximum diameter on axial image or coronal image of NCCT. UWT was defined as the maximum thickness of the ureteral wall at the stone site on axial NCCT image. 3D-MSD was calculated as previously reported [15]. Briefly, 3D-MSD was automatically calculated using Aquarius iNtuition Viewer (TeraRecon Inc., USA), which allows physicians to perform advanced three-dimensional analysis of medical images. The degree of hydronephrosis was graded according to the SFU grade: (0) normal; (1) urine barely splits the sinus; 2) full pelvis, major calyces dilated; (3) uniformly dilated minor calyces, parenchyma spared; and (4) parenchymal compromise [16]. The AH and the RPW were measured using Aquarius iNtuition Viewer (Fig. 1). The long axis of the kidney was set by sagittal image in the slice where the renal pelvis is most dilated. A modified coronal image was created by correction using the long axis. The AH was measured centrally from the line, bordered by the medial edges of the upper and lower poles of the renal parenchyma in a modified coronal image. RPW was defined as the length of the intersection of this line with the renal pelvis. NCCT measurements were evaluated at the abdominal window by expert urologists blinded to the outcome of this study. Impacted stones were defined as those fixed to the ureteral wall that were shown not to move by means of ureteroscopic manipulation or irrigation pressure in the intraoperative videos. Surgical outcomes included operation time, ureteral injury and stone-free rate. Ureteral injury was assessed by the grade described by Traxer and Thomas [17]. ‘Stone free’ was defined as any residual stones being < 4 mm according to assessment by NCCT or KUB within 3 months postoperatively [2, 18, 19].

### Surgical procedures

All URSL were performed in lithotomy position under general anesthesia. First, a semi-rigid ureteroscope (7.5Fr, Karl Storz, Tuttlingen, Germany) was used to observe the ureter and stones, and a guidewire was inserted. Second, a ureteral access sheath (Cook Medical, Bloomington, IN, size; 9.5/11.5Fr, 12/14Fr and Boston Scientific, Marlborough, MA size; 11/13Fr) was placed in almost all patients, except those with U3 stones. Flexible ureteroscope (URF-P6 or P7, OLYMPUS, Tokyo, Japan) was used when an access sheath could be placed. A 270 μm Medilas H Solvo holmium laser (Dornier, Lindau, Germany) was inserted to disintegrate the target stones with an energy level of 0.6–1.2 J and a rate of 8–10 Hz. A 1.5Fr N-Circle nitinol tip-less basket (Cook Medical) was used for stone removal. After completion of stone removal, the ureter was observed by endoscopy to ascertain whether or not there was a ureter injury. Finally, a ureteral stent was used for the patients among whom access sheaths could be placed. Ureteral stent were removed 1–2 weeks after surgery.

### Statistical analysis

Patients’ characteristics, clinical factors and operative factors were compared by chi-square test for categorical data and Mann–Whitney* U* test was used for continuous data. The diagnostic accuracy for the prediction of impacted stones was compared by using ROC curve analyses. Univariate and multivariate logistic regression analyses were performed to identify predictive factors associated with impacted stones. Multivariate logistic models were compared using the Akaike information criterion (AIC). The model with the smallest AIC was considered to be the most appropriate. The identified predictive factors were made into categorical variables by optimal cut off from ROC curves analyses and we examined the risk of impacted stones.

Two-sided *P* values were used, with significance set at 0.05. Data analyses were performed using JMP software version 13 (SAS Institute, Cary, NC, USA).

## Results

The present study included 160 patients treated with URSL. They comprised 54 patients with impacted stones (33.8%) and 106 patients with non-impacted stones (66.2%). Patient characteristics, stone parameters and surgical outcomes are shown in Table 1. Patient characteristics were not significantly different between the two groups. Regarding stone parameters, stones were larger in the patients with impacted stones than in the patients with non-impacted stones (*P* < 0.01). UWT was also thicker in patients with impacted stones than in patients with non-impacted group (*P* < 0.01). The patients with impacted stones had significantly higher SFU grade, larger AH and thicker RPW than the patients with non-impacted stones (all *P* < 0.01). Regarding surgical outcomes, operation time was significantly longer in the patients with impacted stones (*P* < 0.01) and ureteral injuries occurred more often in the patients with impacted stones than in the patients with non-impacted stones (*P* = 0.03). Stone free rate did not differ between the two groups.

**Table 1:** Comparison of patient characteristics, stone parameters and surgical outcome

	Impacted	Non-impacted	*P* value
No. pts	54	106	
Age, years	66.5 (57.8–73.3)	68 (58.8–76.3)	0.28
Male, *n* (%)	33 (61.1)	70 (66)	0.53
BMI, kg/m^2^	23.3 (21.1–26.2)	23.6 (21.0–26.5)	0.81
ECOG PS ≧ 2, *n* (%)	7 (13.0)	23 (21.7)	0.18
Right, *n* (%)	22 (40.7)	51 (48.1)	0.42
Preoperative drainage, *n* (%)	23 (42.6)	46 (43.4)	0.92
Stone size, mm	9.2 (7.1–11.9)	7.7 (5.5–10)	< 0.01
MSD, HU	438 (360–551)	414 (307–500)	0.11
UWT, mm	3.4 (2.8–4.4)	2.9 (1.9–4.0)	< 0.01
Stone location, *n* (%)			0.51
Proximal	34 (63)	61 (57.5)	
Middle/Distal	20 (37)	45 (42.5)	
Grade of Hydronephrosis (SFU), *n* (%)			< 0.01
0	1 (1.9)	22 (20.8)	
1	1 (1.9)	8 (7.5)	
2	10 (18.5)	23 (21.7)	
3	35 (64.8)	48 (45.3)	
4	7 (12.9)	5 (4.7)	
RPW, mm	18.0 (12.3–24.5)	12.1 (5.3–18.5)	< 0.01
AH, cm^2^	13.5 (10.1–16.1)	7.6 (3.7–11.8)	< 0.01
Operation time, min	91 (68–122.5)	62 (43–83.3)	< 0.01
Ureteral injury, *n* (%)	4 (7.4)	1 (0.9)	0.03
Stone free, *n* (%)	48 (88.9)	97 (91.5)	0.59
Stone composition			0.72
CaOx/CaP	49 (90.7)	92 (86.8)	
UA	3 (5.5)	7 (6.6)	
Struvite	1 (1.9)	3 (2.8)	
Others	1 (1.9)	4 (3.8)	

Continuous variables are shown in "median (quartile)" form

*BMI* body mass index, *ECOG PS* Eastern Cooperative Oncology Group Performance Status, *MSD* mean stone density, *UWT* ureteral wall thickness, *RPW* renal pelvic width, *AH* area of hydronephrosis

**Fig. 2:**
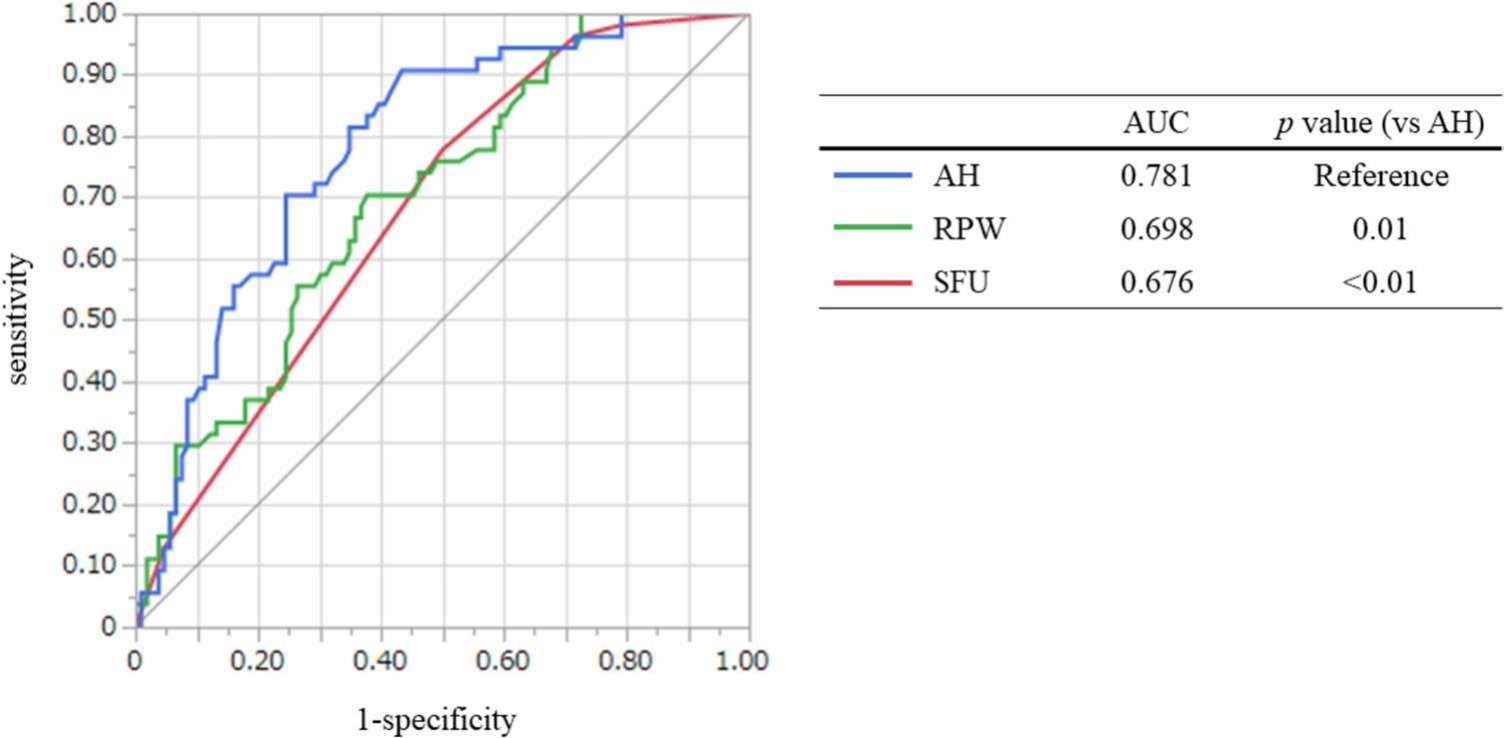
ROC curve analysis for predicting impacted stones

To compare the value for the prediction of impacted stones, ROC curves were analyzed for SFU, the AH and the RPW (Fig. 2). The AH had a significantly higher AUC (0.781) compared with those of RPW and SFU (*P* = 0.01 and *P* < 0.01, respectively).

**Fig. 3:**
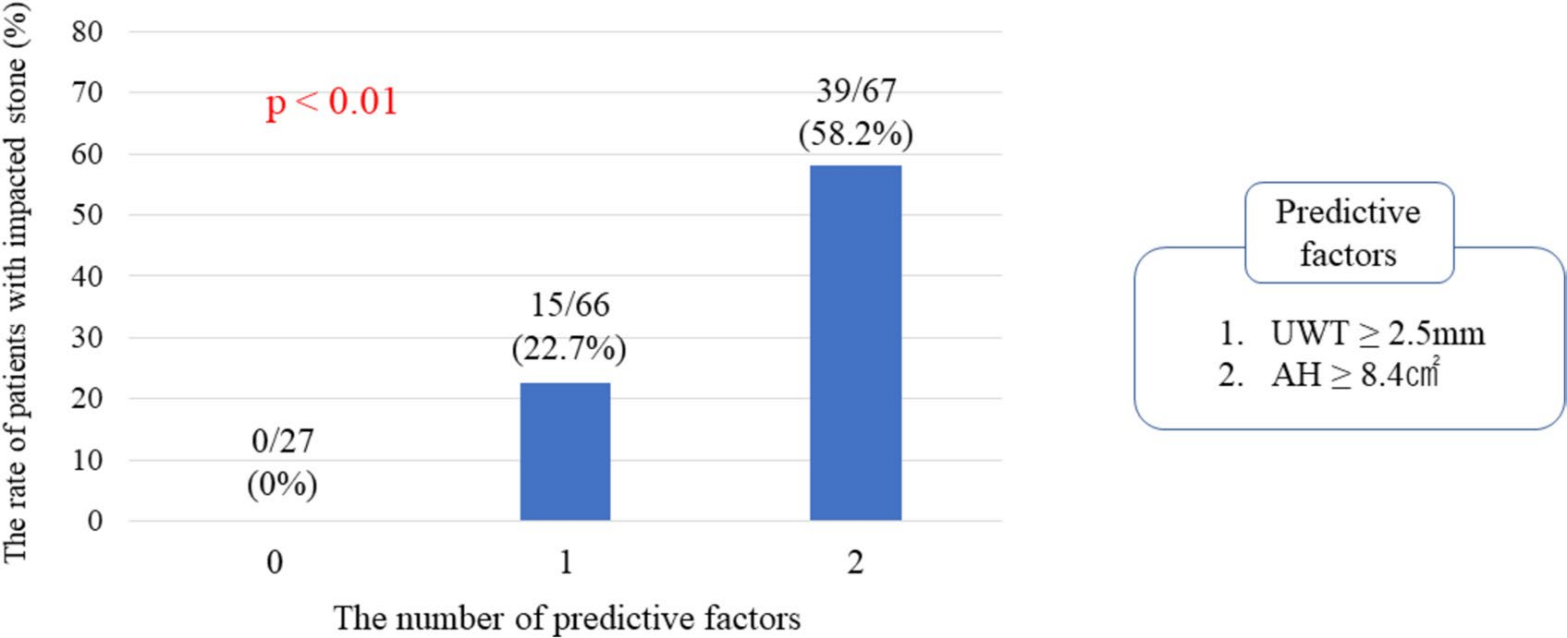
Association between the rate of the patients with impacted stones and the number of predictive factors

In univariate analyses (Table 2), UWT and the AH were significantly different (both *P* < 0.01). Multivariate logistic modes revealed that in addition to thicker UWT, larger AH, RPW and SFU were independent predictive factors for impacted stones in each model (Table 2). The model including the AH had the lowest AIC and was considered to be an appropriate model (AIC: 178.6). The AUC of the combined model (AH and UWT) was 0.784 and slightly higher than AUC of the AH alone. Comparison of the number of predictive factors is shown in Fig. 3. The patients without impacted stones had no risk factors. Conversely, 58.2% of patients with two risk factors had impacted stones.

**Table 2:** Univariate and multivariate logistic regression analyses associated with impacted stone

Variable	Univariate analysis			Multivariate analysis (model 1)			Multivariate analysis (model 2)			Multivariate analysis (model 3)		
	OR	95% CI	*P* value	OR	95% CI	*P* value	OR	95% CI	*P* value	OR	95% CI	*P* value
Age	0.98	0.96–1.01	0.25									
Male	1.23	0.63–2.44	0.54									
BMI	0.98	0.92–1.06	0.66	AIC: 178.6			AIC: 186.6			AIC: 186.7		
ECOG PS ≥ 2	0.53	0.21–1.35	0.17									
Preoperative drainage	0.96	0.49–1.88	0.92									
Stone size	1.07	0.98–1.17	0.08	1.00	0.91–1.12	0.87	1.00	0.91–1.12	0.87	1.00	0.91–1.11	0.86
MSD	1.00	0.99–1.00	0.06	1.00	0.99–1.00	0.13	1.02	0.99–1.00	0.13	1.00	0.99–1.00	0.12
UWT	1.31	1.06–1.62	< 0.01	1.40	1.10–1.78	< 0.01	1.32	1.10–1.78	< 0.01	1.35	1.06–1.71	< 0.01
AH	1.14	1.07–1.21	< 0.01	1.14	1.07–1.21	< 0.01						
RPW	1.10	1.05–1.15	< 0.01				1.09	1.04–1.14	< 0.01			
SFU	2.18	1.45–3.28	< 0.01							2.08	1.35–3.21	< 0.01

*BMI* body mass index, *ECOG PS* Eastern Cooperative Oncology Group Performance Status, *MSD* mean stone density, *UWT* ureteral wall thickness, *APD* anterior–posterior diameter, *AH* area of hydronephrosis, *RPW* Renal pelvic width, *SFU* Society for Fetal Urology, *AIC* Akaike Information Criterion

## Discussion

We evaluated the AH as a predictive factor for impacted stones. The AH was identified as the best predictive factor among hydronephrosis-related factors when compared by AUC analysis. Moreover, the UWT and the AH were preoperative factors to predict impacted stones in multivariate analyses. As risk factors (AH, UWT) increased, there was increase in the rate of patients with impacted stones.

Some predictive factors for impacted stones have been reported, among which UWT is a frequently reported factor [1, 2, 7, 11, 12]. Sarica et al. showed that higher UWT decreases the success rate of shock wave lithotripsy, because UWT is closely related to the degree of impaction [11]. High UWT was shown to be associated with the presence of ureteral edema, polyps, white lesions, stone fixation, longer operation time, and lower endoscopic stone-free rate [7]. The UWT was also determined as a predictive factor for the success of ureteral stent insertion with obstructing ureteral calculi [20]. Impacted stones cause inflammation in the ureteral mucosa and fibrosis of the interstitium. This chronic inflammation can lead to thickening of the ureteral wall, mucosal edema, polyps and stone fixation. Poor endoscopic images make it difficult to disintegrate and remove stones performing URSL. Intraoperative complications were, therefore, reported to be high in impacted stones [6]. In this study, the risk of ureteral injury was higher in patients with impacted stones compared with in patients with non-impacted stones (7.4% vs 0.9%,* P* = 0.03). The UWT was also determined as an independent predictive factor of impacted stones.

In the case of impacted stones, the degree of hydronephrosis is expected to worsen as the degree of obstruction increases because the impacted stone can strongly obstruct the ureter. Hydronephrosis had been reported as a predictive factor of impacted stones [2, 12, 13]. However, these previous papers assessed hydronephrosis qualitatively using a grading system, and there have been no reports in which hydronephrosis was assessed *quantitatively*. A problem with evaluating hydronephrosis by grading system is inter-observer error. Han et al. reported lower Kappa values for SFU grades 2 and 3 compared with Grade 1 and 4 when comparing hydronephrosis by renal ultrasound images [21]. The difference between SFU grade 2 and 3 is whether all renal calyces are dilated, but judgement of dilation is dependent on the observer, leading to lower agreement rate [16]. The AH, meanwhile, can be evaluated quantitatively. In the present study, we believe that the predictive ability was high because the dilation of renal calyces can also be quantitatively evaluated. An important issue with measuring the AH is that NCCT coronal images alone cannot evaluate multiple renal calyces because hydronephrosis is evaluated in a one-slice image (Fig. 1). However, by correcting for the long axis of the kidney as in the present study, multiple renal calyces can be evaluated in a one-slice image, and the AH can be measured more accurately. This method, therefore, enabled us to predict for impacted stones. To the best of our knowledge, this is the first report to quantitatively evaluate hydronephrosis in NCCT when predicting impacted stones. RPW, one of the hydronephrosis-related factors, was significantly different between patients with and without impacted stones when comparing patient characteristics, but was inferior to the AH in AUC analyses. RPW could evaluate dilation of the ureter and reflect the degree of impaction, but the RPW could not be used to evaluate dilation of renal calyces, and this would be the cause of inferiority to the AH.

The AH and the UWT were the independent predictive factors, and the number of predictive factors were associated with the risk of impacted stones (Fig. 3). A positive predictive value of 58.2% is certainly not high, but the negative predictive value was as high as 100%. This risk classification is also useful in terms of ruling out impacted stones. If it can determine that a patient does not have impacted stones, it is more likely that the surgery can be performed safely. Moreover, if impacted stones can be predicted, it may be possible to plan certain treatment strategies preoperatively. Anan et al. showed that preoperative percutaneous nephrostomy would improve the stone-free rate in patients who underwent URSL for impacted stones [14]. Other papers also revealed that antegrade URSL in large impacted upper ureteral calculi was a safe and efficient treatment option [9, 10]. It could also explain that the patient may require multiple treatments if there is a high risk of impacted stones. For these reasons, the ability to preoperatively predict impacted stones is very important for clinicians.

This study has some limitations. First, it was retrospective design and was based on a relatively small cohort in a single institution. In the future, the number of cases should be increased and external validation will be carried out to confirm whether the present study is correct. Second, the AH was evaluated by one slice image. Different slices are expected to measure differently. Ideally, if the volume of hydronephrosis could be measured, it would be possible to assess the whole hydronephrosis precisely, but this would likely take a lot of time. In this study, an AH measured by modified coronal image of NCCT took just a few minutes, and it would be useful in daily clinical practice. If 3D software becomes available in the future that can easily measure the volume of hydronephrosis, it would be useful to compare 2D and 3D hydronephrosis. Third, it is expected that there is some inter-measurer error. Demonstration of the reproducibility of this measurement method is therefore necessary, but the AH and RPW are simple and easy to measure and reproducibility is expected to be high. Fourth, the definition of impacted stones was based on the endoscopic findings. Several definitions of impacted stones have been reported, but there is not yet a fixed definition. Although the definition of a guidewire passage in the first attempt is often used, it is difficult to evaluate retrospectively. Therefore, similarly to other reports, we defined impacted stones based on the endoscopic findings [2, 6]. Despite these limitations, the AH and the UWT were considered to be useful predictive factors for impacted stones.

## Conclusion

AH measured by modified coronal image of NCCT is very useful predictive factor related to hydronephrosis for impacted stones. A combination of the AH and the UWT can thus be an excellent predictor of impacted stones. Predicting impacted stones is thought to be beneficial in surgical planning and preoperative settings.

## Data Availability

The datasets generated and analyzed during the current study are available from the corresponding author on reasonable request.

## Abbreviations

AIC: Akaike information criterion
AH: Area of hydronephrosis
NCCT: Non-contrast computed tomography
RPW: Renal pelvic width
SFU: Society for Fetal Urology
URSL: Ureteroscopic lithotripsy
UWT: Ureteral wall thickness
3D-MSD: Three-dimensional means stone density
